# Harmonising the scope of practice for genetic counsellors in the D-A-CH region: a cross-border consensus for Germany, Austria, and Switzerland

**DOI:** 10.1038/s41431-026-02188-6

**Published:** 2026-07-18

**Authors:** Gunda Schwaninger, Kathrin Taxer, Wera Hofmann, Christophe Cordier, Camila Gabriel, Johanna Tecklenburg, Johannes Zschocke, Simone Heidemann

**Affiliations:** 1 Institute of Human Genetics, Medical University of Innsbruck, Innsbruck, Austria; 2 German Society of Human Genetics Genetic Counsellor Commission Members, Berlin, Germany; 3 Eurofins Humangenetik und Pränatal-Medizin MVZ GmbH, Munich, Germany; 4 Genesupport/Medisyn - Sonic Suisse SA, Lausanne, Switzerland; 5 Institute of Human Genetics, Heidelberg University Hospital, Heidelberg, Germany; 6 Bioscientia Institut für med. Diagnostik GmbH, Ingelheim, Germany; 7 Institute of Tumour Genetics North, Kiel, Germany

**Keywords:** Genetic counselling, Human behaviour

## Abstract

Rapid integration of genomics into clinical practice has increased the demand for genetic counselling services. While the genetic counsellor (GC) profession is well established in the Anglo-American healthcare systems, the D-A-CH countries, Germany, Austria, and Switzerland, operate under distinct, physician-led legal frameworks. To ensure high-quality care and professional mobility, a harmonised definition of the GC's role is necessary. The newly founded GfH Commission for GCs, in collaboration with the national human genetics societies of Germany (GfH), Austria (ÖGH), and Switzerland (SGMG), the Association of German Human Geneticists (BVDH), and the Swiss Association of GCs (ASCG), conducted a multi-stage consensus-building process. The methodology involved a comparative analysis of national legal prerequisites and the application of the entrustable professional activities framework to define the interprofessional interface between GCs and medical geneticists (MGs). The resulting scope of practice (SoP) categorizes competencies across three clinical phases: pre-counselling preparation, the active counselling process, and post-analytical follow-up. The SoP distinguishes between independent GC tasks, shared clinical responsibilities, and tasks requiring MGs. Key to this model is the preservation of the legal physician-prerogative for aspects like the final medical diagnosis and test-ordering while maximizing the GC’s contribution to patient-centred communication and care. This cross-border consensus represents a landmark in European professional policy, providing a scalable blueprint for the integration of GCs into regulated medical systems. By harmonising standards across the D-A-CH countries, the SoP ensures quality assurance, provides a robust evidence base for future legislative recognition and reimbursement of GCs, and facilitates professional mobility in Europe. Genetic counsellor, professional policy, D-A-CH region, interprofessional collaboration, entrustable professional activities, scope of practice, Genetische Fachberater, GfH, ÖGH, SGMG, BVDH, ASCG

## Introduction

The rapid integration of genomic medicine into clinical practice has increased the demand for specialized genetic counselling services. While the Anglo-American model in which genetic counsellors (GCs) are working quite independently is well established, the GC professionals in the D-A-CH countries, Germany, Austria, and Switzerland, operate under distinct, physician-led legal frameworks with a heterogeneous scope of practice among GCs in different genetic centers in the three countries. The French-speaking part of Switzerland has been employing foreign-trained GCs for the last two decades, but the rest of the country—similar to Germany and Austria—only introduced the profession with the first graduates from the MSc course in Genetic and Genomic Counselling at the Medical University of Innsbruck in 2022. This course was established in 2019 and is still the only GC training program in the D-A-CH region [1, 2]. It trains GCs for the entire region, as medical genetic services are closely related by common language and a fluidity of workforce between the countries [3]. While there is still a high demand for genetic professionals, the integration of GCs across Europe varies [4]. Only some European countries, particularly the UK and France [5, 6], have formally integrated the GC profession into their healthcare systems [7, 8].

In 2024, the GfH (German Society of Human Genetics, *Deutsche Gesellschaft für Humangenetik*) established a Genetic Counsellor Commission (*GfH Kommission Genetische Fachberater:innen*) to support the smooth integration of the healthcare profession into existing medical genetic services. Commission members soon realized a need for professional definition, particularly at the interface of medical geneticists (MGs) and GCs. Therefore, the GfH GC commissions’ priority was to develop a scope of practice (SoP) for the GC profession, as it seemed clear that a harmonised definition of the GC role was necessary to ensure high-quality care and effective collaboration in interprofessional genetics teams. While all three countries have hierarchical physician-led medical systems in common, they do have distinct national legal frameworks (Table 1).

**Table 1:** Summary of the three D-A-CH countries, Germany, Austria, and Switzerland, listing the national academic/professional societies and legal frameworks.

Country D-A-CH	Academic Society	Professional Association	Legal Framework
**Germany (D)**	GfH German Society of Human Genetics, Deutsche Gesellschaft für Humangenetik	BVdH Association of German Human Geneticists, Berufsverband Deutscher Humangenetiker	GenDG Genetic Diagnostics Act
**Austria** **(A)**	ÖGH Austrian Society of Human Genetics, Österreichische Gesellschaft für Humangenetik		GTG Genetic Engineering Act
**Switzerland (CH)**	SGMG Swiss Society of Medical Genetics, Schweizerische Gesellschaft für Medizinische Genetik	ASCG Swiss Association of Genetic Counsellors, Association Suisse des Conseillers en Génétique	GUMG Federal Act on Human Genetic Testing

The SGMG serves both as the academic society and the professional association in Switzerland.

In Germany, genetic counselling is strictly regulated by § 7 (3) of the Genetic Diagnostics Act (GenDG) [9]. While the law maintains a firm physician prerequisite, it includes a provision that allows the involvement of non-physician experts (like GCs), provided the counsellee gives their consent. This framework currently limits the independence of GCs, who must work under medical supervision. The vague description of the involvement of non-medical experts in genetic counselling led to uncertainty in the practical implementation of the law.

Austria’s Genetic Engineering Act (GTG) follows a similar physician-led model under § 69 [10]. While the law mandates that a physician perform the consultation, it also suggests the ‘advisability of additional counselling by a psychotherapist or social worker. Currently, GCs are not explicitly named in the statute—mainly because the profession did not exist in Austria when the law was enacted in 1994. Revision of the Austrian GTG may present an opportunity to formalize and determine the role of GCs. Legal protection of professional title and legal regulation as a healthcare profession are absent in both Germany and Austria.

Switzerland adopted a more flexible approach following the 2022 revision of the Federal Act on Human Genetic Testing (GUMG). The revision replaced the strict ‘physician requirement’ with the broader term ‘qualified person’ (German: Fachkundige Person) in Article 21 [11], thereby including GCs. However, formal recognition of the GC profession and reimbursement remain hurdles. GCs are primarily recognized as healthcare professionals in the French-speaking canton of Vaud, but there is no protection of the professional title. Furthermore, reimbursement for genetic counselling services across Switzerland is generally restricted to medical doctors. The definition of a clear SoP could be the basis of a collaborative delegation model for GCs across all three countries.

The original plan to negotiate a SoP focused on the GfH GCs commission and the board of the GfH, but during the iterative consensus process, a unique opportunity arose because, in parallel, the SGMG (Swiss Society of Medical Genetics, *Schweizerische Gesellschaft für Medizinische Genetik*) was working on an SoP for GCs in Switzerland. Therefore, the GC commission engaged with the SGMG to join forces. One by one also the ÖGH (Austrian Society of Human Genetics, *Österreichische Gesellschaft für Humangenetik*), the ASCG (Swiss Association of Genetic Counsellors, *Association Suisse des Conseillers en Génétique*), and the BVDH (Association of German Human Geneticists, *Berufsverband Deutscher Humangenetiker*) became part of the SoP development process and the ratification of the final document, that is composed of an official text in the journal of the GfH [12] and a comprehensive list of entrustable professional activities (EPAs) published on the societies websites (e.g. https://www.gfhev.de/).

## Methods

The development of the SoP for GCs in the D-A-CH region was conducted via a structured, multi-stage consensus-building methodology between October 2024 and January 2026. This process was led by the GfH GC Commission. The process was initiated with a moderated consensus workshop. The session included 85 representatives from 31 university and private human genetic centers across Germany, Austria, and Switzerland. This initial engagement resulted in the definition of the core objectives to pave the way to state recognition of GCs as a regulated healthcare profession, identifying the formalization of a professional SoP as the strategic priority to address the integration of GCs into existing human genetic services in D-A-CH (Schwaninger et al. 2026, JGC under review).

The drafting phase involved a review of international professional standards, primarily the UK framework [13], Australian guidelines [14], and empirical data regarding global GC task distribution [15], as in these countries the GC profession has been well established over decades. Given the structural divergence between Anglo-American healthcare systems and the D-A-CH physician-led model, the framework was iteratively adapted to ensure compliance with national laws (GenDG, GTG, and GUMG). The EPA framework was utilized as the conceptual foundation to define the interprofessional interface, allowing for a scalable delegation model based on professional experience, the EBMG (European Board of Medical Genetics and Genomics) registration status [16], and the practical contributions of the three genetics professions to the interprofessional team (MGs, GCs, and clinical laboratory geneticists (CLGs)) [17, 18].

The initial document was put together by the GfH GC commission and subjected to a reiterative review process. The draft was shared with the boards of all five participating organizations consecutively (GfH, SGMG, ÖGH, ASCG, BVDH). Feedback was gathered through formal written commentary, focusing on four key areas: professional competency, clinical feasibility, legal alignment, and national healthcare specificities. Proposed modifications were reconciled in the GfH GC Commission, followed by discussions between the speaker of the GfH Commission and executive representatives from the partner societies and associations. Changes were then shared among participants in this process to ensure a unified consensus. These iterative steps ensured broad endorsement and readiness for clinical implementation across both academic and private sectors in the D-A-CH countries.

## Results

The SoP resulting from the reiterative consensus process categorizes competencies across three clinical phases: pre-counselling preparation, the active counselling process, and post-analytical follow-up (Box 1).

The SoP is accompanied by a detailed list of tasks that comprises thirteen defined domains with subtasks of the genetic consultation (full list see supplement 1). While the entire genetic consultation is defined to be under the legal responsibility of an MG, the SoP distinguishes between independent GC tasks, shared clinical responsibilities that need approval from the MG, and tasks always requiring an MG (Box 2). The details of this document were developed with the boards of GfH, SGMG, ÖGH, ASCG, and BVDH to bring in all national perspectives, introduce amendments, and achieve broad consensus on the GC tasks within the interprofessional team (details in the methods section). The goals of the document were the maximization of GC contribution to patient-centered communication and care while respecting national legal frameworks, particularly the physician prerogative, and to lay out a clearly defined SoP to promote the integration of GCs within human genetic teams.

GCs provide essential expertise to the interprofessional genetics team by integrating patient-centered communication and psychosocial expertise into clinical routines. GCs utilize their special training that is standardized across Europe by the EBMG [16] to support patients with consideration of health beliefs, individual needs and values, concerns, and other factors, at the outset of the counselling process. By gathering nuanced data on cultural backgrounds, lifestyle, and personal values, they tailor complex genetic information into an accessible format for the patient. While GC practice spans all thirteen SoP domains, GCs often contribute most independently to psychosocial assessment and support—confidently applying communication strategies, exploring resilience and coping, using participatory counselling techniques, facilitating family communication, and linking patients to appropriate resources—thereby strengthening the patient-centered focus of the genetics service. Establishing and implementing a clear, jointly endorsed SoP clarifies which tasks can be delegated, supports role acceptance in interdisciplinary teams, and underpins subsequent steps such as remuneration models and billing codes for GC services. In practice, this structured delegation can expand consultation capacity and quality and streamline workflows, provided there is effective collaboration and trust between GCs and MGs.

The provided SoP gives a detailed list of EPAs that both GCs and supervising MGs can follow in their daily practice, supporting the efficient integration of the GC profession into existing genetic services. Shown in Box 2 is a clear distinction between the physician professional activities and the GC professional activities, which helps to illustrate that there are also areas that are not carried out by GCs and others that can be delegated to GCs to be carried out independently or together with the MGs. The shared activities can strengthen the collaboration of GCs and MGs in the interprofessional team and emphasize that both professions can contribute their strengths to patient-centered care.

Box 1. Key subtasks in the SoP are defined in a linear clinical timeline with three categories

1. Activities prior to genetic counselling and prior to genetic analysis, with a focus on case preparation
2. Activities during the genetic consultation, prior to genetic analysis/prior to clinical genetic diagnosis
3. Activities post genetic analysis/post clinical genetic diagnosis

Box 2. Distribution of professional activities and responsibilities (full list see supplement 1)

1. **GC professional activities—tasks that can be performed by the GC independently (within the team structure) focus on process, communication, and psychosocial support**
  **Preparation and research:** Reviewing medical records, obtaining previous findings, and conducting literature research
  **History and pedigree:** Structured recording of medical history and the creation/analysis of a pedigree spanning at least three generations
  **Psychosocial assessment:** Identifying the emotional situation (e.g. stress, family dynamics, and resilience)
  **Counselling support:** Explaining basic genetic concepts, inheritance patterns, and providing decision-making support based on patient values
  **Documentation:** Drafting the genetic counselling letter and managing communication regarding the status of analyses
2. **Interprofessional activities—these tasks are delegated to the GC but sometimes require medical oversight or direct collaboration**
  **Risk assessment:** Selecting risk models (e.g., Bayes, empirical data) and calculating specific risks for the patient and relatives
  **Test strategy:** Identifying the most informative family members for testing and selecting appropriate laboratory analysis methods
  **Interpretation of results:** Interpreting genetic variants and their clinical significance for the patient’s disease management
  **Long-term planning:** Coordinating follow-up measures and managing multi-generational family information
3. **Physician professional activities—under the legal frameworks (GenDG, GTG, GUMG), certain responsibilities remain with** **thephysician**
  **Physical examination:** All clinical physical examinations are reserved for medical doctors
  **Final medical responsibility:** Verifying and releasing medical indications, final clinical diagnoses, and specific medical management recommendations
  **Legal authorization:** Ordering the genetic analysis and providing the final medical signature/release on diagnostic reports

## Discussion

The development of a joint SoP for the GC profession in the D-A-CH region is professionally and strategically superior to separate national documents, as it establishes a harmonised professional identity beyond political borders while respecting linguistic and cultural overlap in the three countries. Pooling the expertise of three major scientific societies (GfH, ÖGH, SGMG) and professional associations (SGMG, ASCG, and BVDH) from all D-A-CH countries in a consensus is clearly a major achievement compared to isolated national guidelines, especially for the relatively young GC profession in the D-A-CH region. The SoP not only supports the GC profession establishment, but furthermore reduces professional fragmentation, facilitates cross-border workforce mobility, and establishes a robust, evidence-based standard for quality assurance in genomic medicine overall. Ultimately, this joint SoP serves as a strategic blueprint for the formal integration of GCs in academic as well as private genetic services and for legislative recognition of GCs in D-A-CH, ensuring consistent quality in a patient-centered approach.

Despite this harmonisation, challenges remain for the D-A-CH region to reach the professional maturity seen in Anglo-American counselling models. An outlook of strategic priorities for the GC profession and its’ representatives is given in Box 3. The next main aim is to attain formal state recognition or licensure as a healthcare profession for GCs in the D-A-CH countries, which can help to achieve some related goals like the protection of the professional title reimbursement models, and funding of GC positions, supporting institutes and working GCs to facilitate billing and remuneration models. Only when the profession is recognized can the high standards and quality of the GC work be ensured, and the profession can become more visible and attractive within the D-A-CH countries.

Box 3 Future goals for the full integration of GCs in the D-A-CH healthcare systems

**Table:** 

**Future goals for the full integration of GCs in the D-A-CH healthcare systems**	Formal state recognition and protection of the professional title
	A primary hurdle remains the lack of formal recognition of ‘Genetic Counsellor’ (Genetische Fachberater:innen) as a protected professional title in D-A-CH. This document serves as the essential evidence base for future legislative efforts, providing policymakers with a clear definition of the expertise and safety standards inherent in the role. While registration with the EBMG provides an international professional framework, a process of national licensure as a healthcare professional is the final goal for the GC profession
	Sustainable reimbursement models
	Currently, the funding of GC positions often relies on internal department budgets rather than dedicated billing codes. The clear task differentiation provided in the SoP is the first step toward developing specialized reimbursement structures that reflect the value of the specialized professionals
	Scaling the genomic workforce
	As genomic testing expands into most areas of medicine, the GC role must evolve to strengthen medical genetic services for the expanding needs. The D-A-CH model is uniquely positioned to support non-genetics specialists in managing the communication efforts between complex laboratory data, patient understanding, and psychosocial consequences
	Integration with EBMG
	The support of EBMG registration following a defined European curriculum and professional standards for all D-A-CH GCs will ensure that the region remains competitive and that its workforce is mobile across Europe

## Conclusion

The development of the D-A-CH SoP marks a pivotal shift from a physician-centric model toward a structured, interprofessional collaborative framework that represents more than a list of EPAs or a clinical guideline and is catching up with the standards in other countries and the high demand for professionals in genetic services. The defined interface between MGs and GCs can be seen as a foundational step towards a unique professional identity and balances the rigorous legal mandates across three countries with the increasing demand for interprofessional teamwork for patient-centered care in human genetics.

This cross-border consensus represents a landmark in European professional policy, providing a scalable model for the integration of GCs into regulated medical systems across comparable European healthcare systems. By harmonising standards across the D-A-CH countries, the SoP facilitates professional identity and mobility, ensures quality assurance, and could serve as a distinct European path to future legislative recognition of the GC profession.

### What is known about the topic

Germany, Austria, and parts of Switzerland have not yet fully integrated the GC profession. In June 2024, the German Society of Human Genetics (GfH) appointed a commission to promote the professional role of GCs, aiming to improve patient access to genetic services by integrating GCs into existing clinical genetic services within the D-A-CH region. Shortly after the commission’s formation, a consensus-based process was initiated, first engaging 85 genetics healthcare professionals from 31 genetic centers across Germany, Austria, and Switzerland, identifying the need for a robust scope of practice for GCs as a major priority.

### What this paper adds to the topic

The GfH GC commission conducted a structured, multi-stage consensus process integrating the national healthcare specificities discussed with five human genetics societies and associations across D-A-CH region. The framework harmonises professional standards while navigating the distinct legal prerequisites of three countries. Utilizing the “entrustable professional activities” (EPA) model, it defines an interprofessional interface between MGs and GCs. This cross-border agreement provides a strategic blueprint for legislative recognition, facilitating professional mobility and ensuring high-quality, standardized genomic care in Central Europe.

## Supplementary information

List of entrustable professional activities and responsibilities

## Data Availability

The reiterative consensus process produced many versions of the SoP that are stored with the corresponding author. Due to privacy considerations, these drafts cannot be publicly deposited.
