# Supplementary material for: Harmonising the scope of practice for genetic counsellors in the D-A-CH region: a cross-border consensus for Germany, Austria, and Switzerland
Source: Eur J Hum Genet. 2026 Jul 18;34(10):1524–8. doi: 10.1038/s41431-026-02188-6 (PMC13634040; doi:10.1038/s41431-026-02188-6)
Supplement: Supplementary file 1 [file 41431_2026_2188_MOESM1_ESM.pdf]

Scope of Practice for genetic counsellors
in interprofessional cooperation with specialists in human genetics/medical genetics

The clinical activities are divided into three areas:

1. Activities **prior to** genetic counselling and **prior to** genetic analysis with a focus on case preparation
2. Activities **during** the genetic consultation, **prior to** genetic analysis / **prior to** clinical genetic diagnosis
3. Activities **post** genetic analysis / **post** clinical genetic diagnosis

1. Activities **prior to** genetic counselling and **prior to** genetic analysis with a focus on case preparation

Area	Activity	Independent GC tasks	With approval by MG	Tasks requiring MG
**Preparation and preparatory research**	Viewing and reviewing the medical records of the counselee (and the family); obtaining further reports if necessary; indication; verification; management of appointments	x	x	
	Data and literature research	x		
	Preparation of a preliminary evaluation of the consultation and risk assessment with assurance of the medical expertise required for the condition/ indication in question / consultation (e.g. pathogenesis, management options, etc.); Examination of availability, financing basis and organizational requirements of suitable genetic analyses if necessary	x	x	

2. Activities **during** the genetic consultation, **prior to** genetic analysis / **prior to** clinical genetic diagnosis

Area	Activity	Independent GC tasks	With approval by MG	Tasks requiring MG
**Opening of the consulation**	Clarification of the needs and expectations of the counselee, the level of knowledge of the counselee about the disease in question, the ethnic-cultural background, the traditions, the health beliefs, the attitudes, the lifestyle and the values of the counselee	x		
	Explanation of the genetic counselling process; perception of concerns of the counselee; Assessing issues that extend	x		

	beyond the primary reason for the consultation; If necessary, adaptation of the genetic counselling agenda, for example in case of changes during the conversation, in order to specifically address the needs of the counselee			
	If necessary, adjustment of the scope for genetic counselling in the event of newly emerging genetically relevant issues	x	x	
**Taking the individual history**	Structured collection, analysis and documentation of the individual history, previous hospital stays and operations, the current medication, the reproductive and social history, submitted laboratory results and clinical reports with regard to the indication	x	x	
	If necessary, obtain a birth report, view parent/ child pass	x	x	
	Physical examinations			x
**Taking the family history**	Structured collection, analysis and documentation of the family history with age information with regard to the indication; Creation of a pedigree over at least three generations, if necessary with documentation of blood relationships and ethnic origin	x		
	Recording and analysis of emotional reactions of the counselee as well as family dynamics when collecting family history	x		
	Recording of the psychosocial stress and resilience of the counselee (later discussion with the respective responsible specialists), if necessary, offers of psychological support	x		
	Validation of the collected pedigree	x	x	
**Carrying out risk assessments and risk counselling**	Analysis of the pedigree with regard to the indication	x	x	
	If necessary, selection of a risk assessment model (e.g. empirical data, Bayesian analysis, risk assessment models); Carrying out and documenting a risk assessment/risk calculation based on medical, laboratory and genetic information, taking into account the medical, reproductive and family history; if applicable, risk assessment/risk calculation for children and genetic relatives	x		
	Identification of relatives at increased risk for carrying a familial variant	x		
	Counselling for those seeking advice on genetic and non-genetic risk factors and risk modifiers	x		
	If necessary, change of the indication in cooperation with the interprofessional team and/or responsible specialists	x	x	
**Explanation of diagnosis and course of the disease**	Comprehensive explanation of the condition in question on the basis of medical history and family history and guideline/evidence-based facts as well as the needs and expectations of the counselee	x		
	Communication of patient friendly information about the condition in question, basic genetic concepts, etiology/forms of inheritance (explanation of heredity), variable expressivity and penetrance, taking into account the individual situation<br>Communication of<br>- the disease or predisposition in question<br>- basic genetic concepts<br>- etiology/forms of inheritance (explanation of heredity)<br>- variable expressivity and penetrance	x		
	Create/use tools to improve the understanding of the	x	x	

	counselee, e.g. handouts, visual aids, handwritten drawings and other media			
	Discussion of options for prevention, surveillance, therapy, reproduction and clinical management	x	x	
**Explanation and discussion of testing options**	Selection of the most suitable laboratory analysis and laboratory (test options and test strategies, screening, step-by-step diagnostics, research, etc.)			x
	Identification of the most informative individuals for a genetic analysis and, if necessary, in advance of necessary genetic and non-genetic analyses (e.g. immunohistochemistry/MSI analysis in the case of Lynch syndrome)	x	x	
	Explanation of the test options and test strategies as well as potential test results and their relevance as well as personal and familial implications	x	x	
	Discussion of potential costs and financing options (private and public insurance, self-payers), risks, benefits, limitations and alternatives to the chosen genetic analysis	x		
	Support of the counselee in making a self-determined decision with regard to genetic testing and dealing with its effects on the basis of their own values, feelings and experiences, if necessary, mediation of psychological support services	x		
	Answering questions about current data protection regulations and taking into account any concerns of the counselee in this regard, protection of the personal data of the counselee	x		
**Information and consent**	Preparation of the medical information on the basis of a written declaration of consent to be discussed with the counselee	x		
	Obtaining written consent to a genetic analysis (informed consent)			x
	Taking samples (e.g. blood, saliva) for genetic analysis	x		
	Initiation of genetic analyses and, if necessary, further analyses or other measures with medical prerogative			x
	Coordination of genetic analyses, especially in the case of step-by-step diagnostics	x		
	Case presentation in clinical meetings, interdisciplinary tumor conferences and international case discussions, e.g. with experts via CPMS2.0 in the ERN networks, etc.	x	x	
**Ongoing communi-cation**	Clarification of telephone and written questions from doctors and the counselee about the processing status of analyses, inclusion of additional information in the consultation process depending on the indication	x		

3. Activities **post** genetic analysis / **post** clinical genetic diagnosis

Area	Activity	Independent GC tasks	With approved by MG	Tasks requiring MG
**Disclosure of results**	Explanation of findings relevant to the condition in question	x	x	
	Explanation of an unremarkable or inconclusive finding, respectively	x	x	
	Interpretation of the detected gene variant(s); Interpretation and explanation of the clinical significance of the analysis results	x	x	
	Discussion of possible limitations of the analysis results (e.g. sensitivity, specificity, residual risk) in a generally understandable way	x		
	If necessary, identification of further necessary genetic analyses	x	x	
	If necessary, initiation of further necessary genetic analyses			x
	Recommendations for clinical management based on the findings	x	x	
	Explanation of reproductive options	x		
	Identifying difficulties the counselee has in understanding the findings; Recognizing decision tendencies	x		
	If necessary, offer for the counselee to participate in research projects, information on research projects and participation modalities	x		
	If necessary, referral to other specialists			x
	Creation of individualized early detection, prevention, behavior and therapy recommendations for the counselee			x
	Writing of the written human genetic statement (genetic counselling letter) with the signature of the GC	x	x	
	Answering telephone and written inquiries about the consultation letter	x		
**Assessment of the psychosocial situation of the counselee and offer of psychosocial support**	Identifying factors that may influence the counselling interaction	x		
	Assessment of the counselee's resilience and compliance, if necessary, changing the communication strategy according to the reactions. Actively inquire about the resilience and compliance/support of the family/partner	x		
	Recognizing coping strategies, barriers and defense mechanisms, the influence of cultural/religious beliefs and values, social and psychological history	x		
	Communication about emotions, behavior and coping strategies using a participatory counselling technique; Fostering the counselee's competence and autonomy through direct communication and supportive statements	x		
	Support for communication in the family	x		
	Active support in decision-making that is compatible with their values	x		
	Promotion of coping and adaptation strategies	x		

	(compliance/coping and resilience)			
	Provision of external support resources (including arranging contacts with patient associations / patient advocacy groups); Handing out contact information (e.g. business card)	x		
	Recognizing the need for support from other specialists / psychosocial institutions	x	x	
	Support of the core family (counselee and children) and extended family (relatives)	x		
**Follow Up**	Providing written information for the relatives, e.g. "family letters"	x	x	
	Coordination of any necessary medical procedures in connection with the genetic indication	x		
	Consultation with patients for indication-dependent re-presentations	x		
	Answering telephone and written inquiries about the current status of diagnostics and therapy of the respective indication	x	x	
**Follow-Up consultation**	Update of medical, social, reproductive and family history as described in point 2	x	x	
	Documentation of therapies carried out, prophylactic and surveillance measures as described in section 2	x		
	Update of surveillance recommendations as described in section 2	x	x	
	Determination of the current need for early intervention and, if necessary, further diagnostic and therapeutic services as described in section 2			x

Sources:

- Guideline of the Genetic Diagnostics Commission (GEKO) on the requirements for the qualification for and contents of genetic counselling pursuant to § 23 (2) no. 2a and § 23 (2) no. 3 GenDG, publication date 6 December 2023, Federal Health Gazette 2024 · 67:244–254, https://doi.org/10.1007/s00103-023-03822-0
- S2k Guideline Human Genetic Diagnostics and Genetic Counselling, medgen 2018 · 30:469–522, https://doi.org/10.1007/s11825-018-0223-1, published online: 9 January 2019 (guideline is currently being revised)
- Code of professional practice for genetic counsellors in Europe (2010), https://www.ebmg.eu/fileadmin/GCGN_Downloads/EBMGProfessionalAndEducationalStandardsForGeneticCounsellors-July2010.pdf
- International genetic counseling: What do genetic counselors actually do? - Ormond - 2024 - Journal of Genetic Counseling https://doi.org/10.1002/jgc4.1735
- Middleton, A., Taverner, N., Houghton, C. et al. Scope of professional roles for genetic counsellors and clinical geneticists in the United Kingdom. Eur J Hum Genet 31, 9–12 (2023). https://doi.org/10.1038/s41431-022-01214-7
- Human Genetics Society of Australasia - Scope of Practice for Genetic Counsellors, https://hgsa.org.au/Web/Web/Consumer-resources/Policies-Position-Statements.aspx
- Clinical Skills from Internship Case Log_MUI (G. Schwaninger)
- European Board of Medical Genetics (EBMG) https://www.ebmg.eu/408.0.html
- Filges I. et al, Board SGMG: Genetic Counseling: Concepts, Misunderstandings, Perspectives. Swiss Medical Journal 2022; 103(49-50):34-36.
- Position paper of the SGMG on the introduction of the professional profile of a genetic counsellor in Switzerland. 2021. www.sgmg.ch opinions.
- European Board of Medical Genetics (EBMG) https://www.ebmg.eu/408.0.html

Members of the Genetic Counsellors Commission of the German Society for Human Genetics e.V.
Dr. rer. nat. Simone Heidemann, Kiel (chair)
Dr. Gunda Schwaninger, Innsbruck (deputy chair)
Dr. Christophe Cordier, Lausanne
Camila Gabriel, MSc, Heidelberg
Dr. rer. medic. Wera Hofmann, Stuttgart
Kathrin Taxer, MSc, Innsbruck
Dr. med. Johanna Tecklenburg, Ingelheim
Univ.-Prof. Dr. med. Johannes Zschocke, PhD, Innsbruck
